# Atypically Taut Superior Polar Splenic Artery Discovered in a Human Cadaver

**DOI:** 10.7759/cureus.49627

**Published:** 2023-11-29

**Authors:** Peter D Lombardi, Annie Shi Ru Li, Michelle S Sue, Harun S Bola, Danielle C Bentley

**Affiliations:** 1 Faculty of Arts & Science, University of Toronto, Toronto, CAN; 2 Faculty of Medicine, Department of Surgery, Division of Anatomy, University of Toronto, Toronto, CAN

**Keywords:** splenectomies, splenic vasculature, accessory splenic artery, suprapancreatic, spleen, tortuosity, superior polar artery, splenic artery variations

## Abstract

The splenic artery is the largest branch of the celiac trunk and frequently presents with anatomical variability. These variations relate to its origin, trajectory, location relative to the pancreas, terminal branching pattern, and the potential presence of polar arteries. Knowledge of the splenic artery's variability may inform gastrointestinal surgeons as they plan and execute surgical interventions, resulting in improved success rates while minimizing both operative complications and procedural time. The case presentation of a splenic artery dissected from an elderly male cadaver initially demonstrated normal anatomical arrangement. The artery branched off the celiac trunk of the abdominal aorta and followed a tortuous suprapancreatic route to split into two lobar arteries terminating in the spleen. However, upon closer inspection, a superior polar splenic artery was uncovered with two unique characteristics. Firstly, the presented polar artery lacked branching gastric arteries, a rare variation with a prevalence of only 3.27%. Secondly, the distance between the origin of the superior polar splenic artery and the splenic hilum was greater than what is often reported in clinical literature. While similar previous case reports have observed arterial origins of greater distance, these have often been accompanied by a compensatory arterial length. Interestingly, the case presented in this report contained a superior polar splenic artery with an arterial length shorter than its distance to the splenic hilum, resulting in an atypically taut vessel. This bears clinical importance, as this arterial presentation may be susceptible to a surgical rupture if neglected. By including this anatomical variation in the expanding library of splenic artery variations, surgeons and their collaborative healthcare teams may broaden their understanding of splenic artery anatomy as they conceptualize new techniques for pancreatomy and splenectomy procedures that consider arterial variations while minimizing surgical complications, operative time, and patient blood loss.

## Introduction

Splenectomies are the most common surgical intervention for treating splenic ruptures, with approximately 500 performed annually in the Canadian province of Ontario and 22,000 performed annually across the United States [1,2]. However, unexpected anatomical variations of the splenic artery can lead to surgical complications, prolonged operative time, and severe blood loss [3]. The splenic artery is the largest branch of the celiac trunk and frequently presents with variations in origin, trajectory, location relative to the pancreas, and terminal branching pattern [4]. Accessory arteries may also be present, extending from the splenic artery towards the superior and/or inferior poles of the spleen. They are called superior polar and inferior polar arteries, respectively [4]. An understanding of such potential splenic artery variations can guide surgeons during the planning of splenectomies and other gastrointestinal surgeries to reduce the risk of iatrogenic injuries or complications [3,5].

In its typical presentation, the splenic artery travels laterally from the centrally located celiac trunk, posterior to the stomach, along the superior margin of the pancreas, and towards the hilum of the spleen [6]. Branches of the splenic artery provide oxygenated blood to three structures: the pancreas via the dorsal, transverse, and greater pancreatic arteries; the stomach via the short gastric and posterior gastric arteries; and the greater omentum via the left gastro-omental artery [7]. Cumulatively, the splenic artery and its branches are responsible for supplying blood to the left hypochondrium and epigastric regions of the abdomen [6]. The splenic artery terminates at the splenic hilum, where it commonly divides into two splenic lobar arteries to supply the superior and inferior lobes of the spleen [8].

Functionally, the spleen is a secondary lymphoid organ [9], divided into the lymphoid white pulp and hematogenous red pulp. The white pulp contains lymphocyte aggregates and macrophages that surround arterioles, facilitating the exposure, recognition, and activation of blood-borne antigens [9]. The red pulp is highly vascularized and contains macrophages that are crucial in filtering the blood of debris and old or damaged erythrocytes [9].

The spleen's peripheral position in the left hypochondrium of the abdomen puts it at an increased risk of rupture from blunt force trauma. This increased risk, coupled with the spleen's high degree of vascularization, makes splenic ruptures a leading cause of internal abdominal bleeding [10]. To prevent excessive blood loss and alleviate pain, the conventional procedure to remedy a splenic rupture is to perform a splenectomy [10]. During this procedure, the splenic artery and vein are ligated at the hilum of the spleen, and the spleen is surgically removed from the patient [11]. Therefore, it is imperative that surgeons be aware of splenic artery variations, including the potential presence of superior or inferior polar arteries that would require additional ligation. Successful ligation of these accessory structures can prevent disturbances to the arterial supply of the pancreas, stomach, and greater omentum. Despite the obvious benefit of a splenectomy for the control of bleeding, a noteworthy disadvantage is the consequent reduction in immune function and subsequent risk of infection [9].

In response to the shortcomings of the aforementioned procedure, a partial splenectomy is emerging as an alternative treatment option for a splenic rupture [12]. This conservative procedure requires ligation of only the splenic arterial branches supplying the affected splenic lobes while preserving the unaffected splenic lobes and their arterial supply [8]. As such, patients retain considerable immune function, which reduces susceptibility to post-operative infection when compared to other methods [9,12]. However, success of the partial splenectomy remains contingent on a strong understanding of splenic vasculature to ensure minimal blood loss and adequate oxygenation to the retained splenic lobules post surgery [8].

## Case presentation

**Figure 1:**
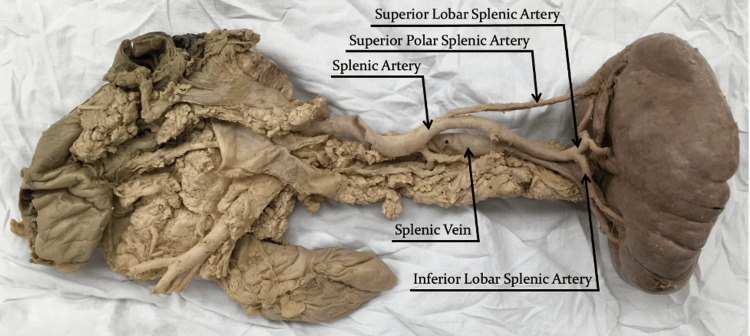
Anterior view of the splenic artery tracing a path towards the splenic hilum.

Students were guided through in-laboratory tasks via Grant's Dissector, 17th Edition [13]. During an educational dissection of the duodenum, pancreas, and spleen on a formalin-fixed 80-year-old Caucasian male cadaveric donor, the presence of a superior polar artery emerging from the splenic artery was discovered. Further investigation of the structure required the removal of the duodenum, pancreas, and spleen complex with careful dissection of surrounding tissue to unveil the accessory structure (Figure 1). No other structural anomalies were observed. The medically documented cause of death for this donor was respiratory failure and metastatic prostate cancer.

**Figure 2:**
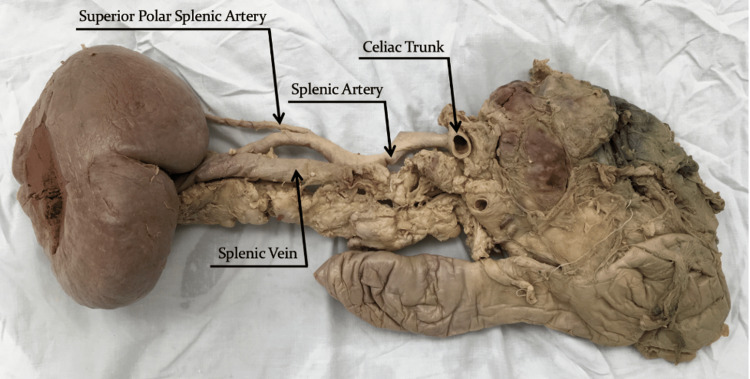
Posterior view of the splenic artery originating from the celiac trunk and travelling laterally along the superior border of the pancreas towards the splenic hilum.

**Figure 3:**
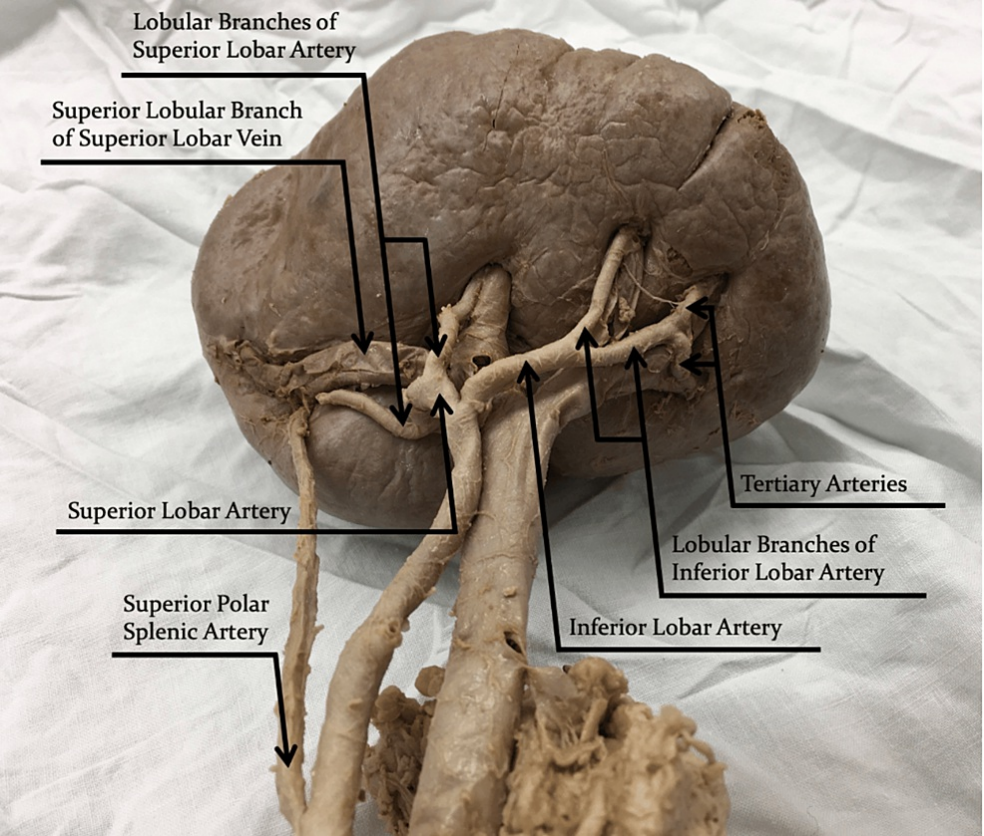
Splenic hilar view of the splenic artery with its branches and the splenic vein with its tributaries.

The splenic artery had a length of 14.6 cm. It originated from the celiac trunk and travelled laterally along the superior margin of the pancreas with a tortuous trajectory towards the splenic hilum (Figure 2). The splenic artery terminated with a distributed branching pattern, including a traditional bifurcation of the splenic artery into superior and inferior lobar arteries 2.1 cm medial to the splenic hilum. The superior lobar artery immediately divided into two lobular branches, whereas the inferior lobar artery travelled 1.8 cm inferiorly before dividing into two lobular branches. The inferior lobular branch of the inferior lobar artery uniquely bifurcated into two tertiary branches before penetrating the spleen (Figure 3).

A superior polar artery was observed originating from the main trunk of the splenic artery 8.0 cm from the splenic hilum and entering the spleen at the superior pole. It was 6.8 cm in length with a diameter of 0.25 cm (Figure 1). Many of the splenic arterial branches were accompanied by concomitant splenic veins. Interestingly, the superior lobular branch of the superior lobar vein did not travel adjacent to its arterial counterpart. Instead, it exited the spleen adjacent to the superior polar artery and travelled 2.6 cm inferiorly to join the superior lobar vein. A posterior view of the specimen (Figure 2) depicts the splenic vein travelling towards the hepatic portal vein. However, the drainage pattern could not be confirmed since the medial portion of the splenic vein and the hepatic portal vein were removed during the preceding dissection task, which had been completed in advance of this exploration.

## Discussion

The splenic artery can be classified based on its presentation in five categories: 1) variations in its origin, 2) variations in the complexity of its path, 3) variations in its location relative to the pancreas, 4) variations in the number of terminal branches and the associated pattern of that branching, and 5) the presence or absence of polar arteries [4]. Classifying the splenic artery based on these five categories can guide gastrointestinal surgeons in their approach to procedures and help them make the necessary adjustments to ensure successful operative outcomes.

Benjamin Lipshutz first proposed that variations in the splenic artery origin can be categorized using a four-type system: Type 1 splenic arteries which are the most common variation, with a prevalence of 75%, originate as a branch off the celiac trunk; Type 2 splenic arteries, with a prevalence of 15%, originate as a branch off the hepatosplenic trunk; Type 3 splenic arteries, with a prevalence of 6%, originate directly from the abdominal aorta; and Type 4 splenic arteries, with a prevalence of 6%, originate as a branch off the splenogastric trunk [14]. With respect to arterial origin, the presented case can be categorized as a Type 1 splenic artery, as it originated from the celiac trunk, best seen in Figure 2.

The splenic artery can also be categorized based on its trajectory from its point of origin towards the splenic hilum, with three variations of increasing complexity: straight, tortuous, or serpentine [4]. Covantsev et al. examined 330 human cadaveric specimens and found that the straight path was the most common, observed in 43.03% of cases [4]. The tortuous path, described as a sinusoidal route towards the splenic hilum, was observed in 27.58% of cases [4]. Finally, the serpentine path, described as extensive looping of the splenic artery, was observed in 20.91% of cases [4]. A fourth variation, entitled alternating, is a combination of more than one of the aforementioned variations and was observed in only 8.48% of cases [4]. With respect to the tortuosity of its path, the presented case can be categorized as tortuous, with the splenic artery's sinusoidal route best visualized in Figure 1 and Figure 2.

A tortuous splenic artery can be clinically advantageous as it prevents blood flow obstruction of the splenic artery during processes such as stomach expansion and respiration [4]. It is hypothesized that tortuosity increases with age due to the splenic artery's lengthening throughout life while remaining anchored to the pancreas via its pancreatic branches [4]. In support of this hypothesis, Sylvester et al. examined 29 human cadaveric specimens and 44 celiac angiograms, and their findings suggested that tortuosity increases with age [15]. In addition, Daisy Sahni et al. were unable to find tortuous splenic arteries among fetuses, infants, or young children [7]. The presented case documents a tortuous arterial trajectory within an 80-year-old donor, further supporting the prevailing hypothesis that the likelihood of tortuosity increases with age. A noteworthy consideration is that, on rare occasions, extensive tortuosity can be associated with a clinical disease pattern resembling chronic pancreatitis [4].

The splenic artery can also be categorized based on its location relative to the pancreas. A systematic review by Manatakis et al. involving the analysis of 3,132 cadaveric specimens across 30 independent studies reported the splenic artery in a suprapancreatic position in 77.4% of cases [12]. Variations of the splenic artery's relative location included a retropancreatic position (17.8% of cases), an anteropancreatic position (3.4% of cases), and an intrapancreatic position (only 1.3% of cases) [12]. The location of the splenic artery relative to the pancreas in the presented case can be categorized as suprapancreatic, with the splenic artery travelling along the superior margin of the pancreas.

Knowledge of the splenic artery's relative location can provide gastrointestinal surgeons with insight into the most appropriate method for approaching procedures beyond splenectomies. For example, a two-type model was recently developed by Wada et al. to determine the approach during laparoscopic distal pancreatomies [5]. In this model, Type S splenic arteries curve and travel in a suprapancreatic position, while Type D splenic arteries travelled straight and dorsal to the pancreas [5]. When the researchers tailored the procedures' approach based on the type of splenic artery, 25 of 30 patients required no unplanned methodological changes, and the researchers claimed this technique improved both surgical efficacy and safety [5].

The fourth category used to classify the splenic artery is the number of terminal branches it has and the associated pattern of that branching [12]. Understanding the intricate relationship between arterial branching and splenic blood flow has been imperative in guiding gastrointestinal surgeons to resect splenic lobes without compromising the health of the retained regions [8]. The number of terminal branches, termed lobar arteries, is two in 86.9% of cases, three in 11.5% of cases, and greater than three in only 1.6% of cases [12]. Multiple studies have confirmed that the number of lobar arteries is directly proportional to the number of independently functional splenic lobes [7,8]. The case presented in this report had the most typical variation of two lobar arteries supplying two splenic lobes.

In addition to the overall number of splenic artery terminal branches, their branching pattern is also clinically significant. Among 72.7% of cases, branching of the splenic artery occurs in a distributed pattern, defined by an early termination of the main arterial segment with subsequent divisions into lobar arteries and lobular branches that penetrate a wide splenic hilum [12]. Alternatively, among 26.9% of cases, branching occurs in a magistral pattern, defined by a late termination of the main arterial segment with the branching arteries penetrating a narrow splenic hilum [12]. The presented case had a distributed branching pattern, with the lobar and lobular arteries entering a wide splenic hilum. The type of branching pattern is an important determinant when predicting splenectomy outcomes. The wide hilum observed in a distributed morphology allows for the easier dissection of the splenic porta and lobes, an advantage for partial splenectomies [12]. Conversely, magistral morphology provides greater control of bleeding during surgery and easier ligation of the splenic artery [12]. This can be beneficial for complete splenectomies but may lead to complications during partial splenectomies due to the increased difficulty of isolating the lobar arteries and associated splenic lobes [12]. Consequently, a patient's arterial branching pattern may influence a surgeon's decision to perform either a complete or partial splenectomy.

Finally, the splenic artery can be categorized based on the presence or absence of accessory arterial branches arising from either the splenic arterial trunk or any one of its major branches [12]. When such accessory branches extend towards either the upper pole or lower pole of the spleen, they are described as superior or inferior polar splenic arteries, respectively [12]. Polar splenic arteries are common, with an estimated prevalence of 41.7% for a superior polar artery and 47.7% for an inferior polar artery [12].

The presented case contained a superior polar artery. In the majority of instances, a superior polar artery will send off additional arterial branches to the inferior aspect of the stomach via posterior gastric arteries [16]. However, the presented case is especially notable as the superior polar artery did not send off any gastric arteries, an observation made in only 3.27% of the population [16]. Furthermore, the length of a splenic polar artery in reference to the distance from its point of origin to the splenic hilum is an additional clinically relevant feature. An investigation by Silva et al. examined 11 splenic polar arteries and found the average distance between the arterial point of origin and the splenic hilum was 4.85 cm [17]. This greatly contrasts with the distance of 8.0 cm described in this case presentation. In such rare cases of increased distance between the polar artery origin and splenic hilum, investigators have observed compensatory longer polar arteries. For example, Baidwan et al. reported a superior polar artery with an origin-to-hilum distance of 6.4 cm and a compensatory arterial length of 7.6 cm [16]. Most interestingly, the superior polar artery described in this case presentation had a length of only 6.8 cm despite the aforementioned 8.0 cm origin-to-hilum distance. The artery, therefore, traced an atypically straight and taut path towards the superior pole of the spleen. While a more fixed position may lead to a higher risk of arterial tearing, there have only been three reported cases of gastrointestinal bleeding attributed to polar splenic arteries [18,19]. Grippi and Yu recently discovered a pseudoaneurysm leading to gastrointestinal bleeding from a superior polar splenic artery in a 73-year-old female patient [19]. While aneurysms or pseudoaneurysms of polar arteries are exceedingly rare pathologies, they are potentially fatal complications that require rapid diagnosis and treatment due to their high morbidity and mortality rates [20]. The length of the patient's superior polar splenic artery was similar at 8 cm; however, its origin was located only 6 cm from the splenic hilum, further highlighting the peculiar morphology of the presented case. It is imperative that gastrointestinal surgeons and their healthcare teams are aware of polar splenic arteries, as failing to acknowledge their presence may lead to unexpected vessel damage during surgery, increased blood loss, and prolonged operative time [16,19]. This may be especially true in instances such as the presented case where the superior polar artery is tightly stretched due to its short length relative to the hilum-to-origin distance.

## Conclusions

This case presentation exhibited the majority of typical anatomical features of the splenic artery, including an origin from the celiac trunk, a tortuous trajectory, a suprapancreatic position, and termination into two lobar arteries with a distributed branching pattern. However, this was a peculiar case worthy of attention due to the presence of a superior polar artery with two distinct features. First, the superior polar artery lacked gastric artery branches, a rare variation found in only a small percentage of the general population. Second, the superior polar artery was short compared to its origin-to-hilum distance, which resulted in an atypically taut artery susceptible to tearing during surgical intervention.

By adding to the existing literature regarding anatomical presentation of the splenic artery, the presented case may help surgeons and their collaborative healthcare teams gain a more comprehensive understanding of potential variations they may encounter during procedures. Variations such as the artery's branching pattern and location relative to the pancreas can have a strong influence on the techniques employed during surgical procedures. Furthermore, the presence of polar arteries is an important consideration during procedural planning to reduce the risk of iatrogenic injuries or complications during the minutiae of gastrointestinal surgeries. Recognition of how splenic artery variations may influence surgical outcomes can enable surgeons to develop appropriate techniques to account for such variations and reduce the potential of unforeseen complications.
